# Anaesthesia for lower-segment caesarean section: Changing perspectives

**DOI:** 10.4103/0019-5049.71037

**Published:** 2010

**Authors:** Sean Brian Yeoh, Sng Ban Leong, Alex Sia Tiong Heng

**Affiliations:** Department of Women’s Anaesthesia, KK Women’s and Children’s Hospital, Singapore

**Keywords:** Caesarean section, obstetrics, regional anaesthesia

## Abstract

The number of caesarean sections has increased over the last two decades, especially in the developed countries. Hence, it has increasingly become a greater challenge to provide care for the parturient, but this has given obstetric anaesthetists a greater opportunity to contribute to obstetric services. While caesarean deliveries were historically performed using general anaesthesia, there is a recent significant move towards regional anaesthesia. Unique problems that patients with obesity and pre-eclampsia present will be discussed in the present article. New medications and devices now used in obstetric anaesthesia will change the practice and perspectives of our clinical practice.

## INTRODUCTION

Obstetric anaesthetists are faced with the unique situation of providing anaesthesia for caesarean sections, where anaesthetists have to provide care for both the mother and the unborn baby. A team approach is vital to ensure optimal outcome while ensuring that the labour process is a safe and pleasant experience for the parturient.

There has been an increasing trend in the caesarean section rate in the last two decades not just in developed countries but also in developing countries. A study in the United Kingdom showed that the rate of caesarean section has increased from 12.5% in 1990 to 18.3% in 1999,[1] while in China, there has been an increase from 8.9% in 1993–1994 to 24.8% in 2001–2002.[2] In our institution in Singapore, the rate has been documented as high as 25.2%.[3] The reason for this rise is multifactorial. A possible reason is an increase in elective caesarean sections due to the preference of patients and obstetricians.[2] An increase in urgent or emergency caesarean sections has been attributed to more advanced intrapartum foetal monitoring, allowing obstetricians to diagnose intrapartum foetal compromise earlier and more effectively.[4]

There has been a move towards more caesarean sections being performed under regional anaesthesia compared to general anaesthesia.[5] New techniques for regional anaesthesia, such as the combined spinal epidural (CSE) anaesthesia and the continuous spinal anaesthesia, offer specific advantages. There has also been recent interest in the use of supraglottic airway devices for caesarean section under general anaesthesia, especially when difficult airway is encountered. Maternal comorbidities such as obesity and pre-eclampsia also present a challenge to the obstetric anaesthetists.

## GENERAL VS. REGIONAL ANAESTHESIA FOR CAESAREAN SECTION

The type of anaesthesia chosen for caesarean section is dependent on numerous factors such as the urgency and indication of the operation, maternal preference as well as coexisting medical problems. The Confidential Enquiry into Maternal and Child Health (CEMACH), which is published every 3 years, has had a great impact on maternal and newborn health over the last 50 years. The number of direct deaths attributable to anaesthesia has dropped significantly since the mid 1980s from the advancement of anaesthetic care. In the latest report that covers maternal deaths from the triennium 2003–2005,[6] only six direct deaths were attributable to anaesthesia. However, in the last two reports, there were a disproportionately greater number of direct deaths associated with general anaesthesia, often related to difficult airway management.[6]

## GENERAL ANAESTHESIA

There are many indications for general anaesthesia, some of which are failed regional anaesthesia, conditions where regional anaesthesia is contraindicated, maternal request and life-threatening foetal compromise[7] when there might not be adequate time to perform a regional technique. In the past, general anaesthesia was considered to be the technique of choice. However, the proportion of caesarean sections performed under general anaesthesia has dropped significantly. In the United States, general anaesthesia is used for less than 5% of elective caesarean deliveries. For emergency deliveries, the rate varies between 15 and 30%.[5] It is not surprising that the experience with general anaesthesia is decreasing, especially among trainee anaesthetists.[8]

Airway problems are more common in pregnancy than in the general population due to anatomical and physiological changes during pregnancy.[9] Some anatomic changes that may affect the obstetric airway include upper airway oedema, breast enlargement and excessive weight gain.[9] A study of obstetric patients has shown an association between difficult intubation and a short neck, obesity, receding mandible and protruding maxillary incisors.[10] It is therefore necessary to ensure that there is a well thought-out difficult obstetric airway algorithm with availability of airway adjuncts to deal with airway emergencies during difficult or failed intubation.

Pulmonary aspiration is one of the concerns of general anaesthesia in obstetric patients. Risk factors for increased risk of aspiration include a prolonged gastric emptying time in labour, increased intra-abdominal pressure due to the gravid uterus and relaxation of the lower oesophageal sphincter due to hormonal changes. To reduce this risk, prophylaxis against acid aspiration is administered prior to anaesthesia.[11] The use of rapid sequence induction with thiopental and succinlycholine has remained standard and largely unchanged for the last four to five decades and was developed to decrease the incidence of pulmonary aspiration.[12] Recent pharmacological development may replace succinylcholine with a high-dose rocuronium–suggamadex combination in the near future.[13,14] Sugammadex, a selective relaxant-binding agent, antagonizes the effects induced by rocuronium on muscle tissue and quickly resolves neuromuscular blockade. Therefore, in situations when a fast onset and short duration of muscle relaxant is required, rocuronium has a reasonably rapid onset and now can be reversed with sugammadex.

## THE USE OF SUPRAGLOTTIC DEVICES FOR GENERAL ANAESTHESIA IN OBSTETRIC PATIENTS

Supraglottic airway devices such as the laryngeal mask airway (LMA) and the LMA Proseal^™^ have been used successfully in obstetric patients undergoing general anaesthesia.[9] The LMA and the LMA Proseal^™^ hold great potential in the management of the obstetric airway. The LMA Proseal^™^ incorporates a second tube intended to permit continuity with the gastrointestinal tract and isolation from the airway, minimizing gastric insufflations during positive-pressure ventilation.[15] The LMA Proseal^™^ has been used successfully as a rescue device during failed rapid sequence induction in obstetric patients,[16,17] Therefore, LMA Proseal^™^ holds great potential to be incorporated in the obstetric difficult airway algorithm in the foreseeable future.

A large prospective cohort study involving 1,067 obstetric patients concluded that the LMA is effective and probably safe in most elective caesarean deliveries.[18] Patient selection is paramount to ensure safety using a supraglottic airway requiring proper airway assessment, adequate fasting and non-obese parturients. An anaesthetist with proficient regular use of LMA is yet another prerequisite. Effective airway was obtained in 99% of the parturients and only seven parturients required intubation. There were no reported episodes of aspiration, gastric insufflations, hypoxia, layrngospasm or bronchospasm. The LMA has also been incorporated into the obstetric difficult airway algorithm. However, the routine use in elective caesarean deliveries is debatable.

## REGIONAL ANAESTHESIA

The proportion of caesarean sections performed under regional anaesthesia has increased greatly over the last two decades, and this has avoided the problem of difficult airway during anaesthesia.[4,5] This is coupled with improvements in the development of safer local anaesthetics, such as ropivacaine and levobupivacaine. The main types of regional techniques used for caesarean delivery are single-shot spinal anaesthesia, epidural anaesthesia and CSE anaesthesia. There have also been documented cases of continuous spinal anaesthesia used for caesarean delivery.

There are obvious advantages of regional anaesthesia, including avoiding the problem of a difficult airway, avoidance of multiple drugs required for general anaesthesia as well as allowing the parturient to be awake to witness the delivery of her baby thus enabling her to participate and enjoy the birthing experience. The Royal College of Anaesthetists in the United Kingdom has proposed that more than 95% of elective caesarean deliveries and more than 85% of emergency caesarean deliveries should be performed using regional anaesthetic techniques.[19]

## SINGLE-SHOT SPINAL ANAESTHESIA

This is by far the most common method of anaesthesia for elective and emergency caesarean sections.[5] The rapidity of spinal onset is especially useful in cases where delivery of the foetus must be expedited due to a compromised foetal state. A case series of 25 patients in the United Kingdom has described the use of spinal anaesthesia in category-1 caesarean section. While case selection is important, anaesthesia can successfully be established in suitable parturients in 6–8 min when using “rapid sequence spinal” anaesthesia. Several components of a rapid sequence spinal have been described, including the “no touch” technique of donning gloves, the omission of spinal opioids while increasing the dose of hyperbaric bupivacaine 0.5% (up to 3 ml) and limiting the number of attempts. Furthermore, one must be prepared to convert to general anaesthesia if the level is inadequate or if other complications arise.[20]

There are several advantages of single-shot spinal anaesthesia. The ability to coadminister analgesics such as opioids allows post-operative analgesia, improving maternal comfort in the post-operative period. Spinal blocks also have the advantage of being more cost-effective when compared with epidural anaesthesia. The difference in cost was attributed to the higher complication rate in epidurals and the significantly longer total operating room times for epidural blocks that tend to take a longer time to establish.[21]

One obvious disadvantage of spinal anaesthesia is the inability to extend the block if the original block height is deemed to be inadequate or if the surgery takes longer than predicted. It is therefore vital to ensure adequate block before commencing surgery as a failure to do so could result in patient discomfort, conversion to general anaesthesia and possible medicolegal implications.

## EPIDURAL

Patients who already have indwelling epidurals for labour analgesia and who subsequently require a caesarean delivery are able to extend their block by giving a “top-up” dose of local anaesthetics. Before augmenting an epidural block, it is vital to ensure that the epidural is functioning well during labour and that no blood or cerebral spinal fluid is aspirated from the catheter prior to giving boluses of local anaesthetics.

Larger doses of local anaesthetics are used and therefore it is important to ensure that the toxic dose of local anaesthetics is not exceeded. Safer local anaesthetic agents are now used, such as lignocaine, ropivacaine and levobupivacaine. Levobupivacaine may play a greater role in the future. Levobupivacine, being the S enantiomer of bupivacaine, is less cardiotoxic than racemic bupivacaine in the event of an accidental intravascular injection.[22]

When spinal and epidural techniques were compared in a systematic review, there was no significant difference with respect to the need for additional intra-operative analgesia, need for conversion to general anaesthesia or maternal satisfaction.[23]

## CSE

This method combines the advantages of the two regional techniques described above. It is able to produce a quick and dense block while allowing an anaesthetist to administer subsequent doses of local anaesthetics via the epidural catheter should the need arise. The epidural catheter can also be used for post-operative analgesia.

This method is especially useful in patients with certain medical conditions, such as high-risk cardiac patients, where it is necessary to titrate the block height carefully. A teaching maternity unit in the United Kingdom recently performed an audit including 3,519 elective caesarean sections using the CSE technique over a 10-year period. The result showed a need for conversion to general anaesthesia of only 0.23%.[24]

This is lower than previous reports of single-shot spinal anesthesia, which has a general anaesthesia conversion rate of 1.2–1.4%.[25,26]

## CONTINUOUS SPINAL ANAESTHESIA

Its popularity declined due to its complications, such as neurologic complications, technical difficulties as well as the potential for post-dural puncture headache. The apparent high risk of post-dural puncture headache had led to the development of microcatheters, which were unfortunately associated with kinking and breakage.[27,28] In the early 1990s, the Food and Drug Authority in the United States banned the use of catheters finer than 24 G due to its possible association with cauda equina syndrome.[29] Nowadays, with the use of newer spinal catheters that have smaller gauges, the interest in this controversial form of regional technique has increased. This technique has the benefits of spinal anaesthesia with the possibility of block extension, with very small doses of local anaesthetics. However, a recent cohort study showed that continuous spinal anaesthesia may be associated with an increased failure rate and post-dural puncture headache.[30]

## OBESE PATIENTS

The incidence of obesity has increased worldwide and occurs with an increasing frequency in the pregnant population.[31] Obesity is now characterized as a pandemic by the World Health Organization. Obesity not only increases maternal risks but also the foetal and neonatal risks.

Obese patients have been found to require more instrumental deliveries and caesarean sections[32] thus requiring more anaesthetic interventions. Obesity has been identified as a risk factor for anaesthetic-related maternal mortality by the last two CEMACH reports. In the last CEMACH report, of the six direct deaths attributable to anaesthesia, four patients were obese and, of these, two were morbidly obese, with a body mass index of greater than 35. Supervision is highly advised for trainees to prevent morbidity and mortality in obese parturients.[6]

Obese parturients often have coexisting medical problems. When faced with an obese parturient, an anaesthetist must look out for the possibility of obstructive sleep apnoea, hypertension, ischaemic heart disease, gastroesophageal reflux as well as diabetes. These diseases are known to be more common in the obese population.[33] It is therefore important to refer these patients for an early anaesthetic consult to plan both labour analgesia and anaesthesia.

The obese parturient also presents practical problems. It is often difficult to find an appropriately sized blood pressure cuff and poses as a problem, especially in regional anaesthesia, where close monitoring of blood pressure is required. Venous access may be difficult and regional anaesthesia may be more technically challenging. Special operating theatre beds must be brought in to ensure adequate weight-bearing capacity.[33] Adequate personnel are required to aid with lifting of patients during transfers from the operating bed to trolleys.

Regional techniques can be challenging in obese parturients due to technical difficulties. Furthermore, surgery may be technically challenging and may take longer than expected.[31,33] The location of the midline for regional techniques may be difficult in the obese parturient. The body anatomical landmarks that are normally palpable may be obliterated by adipose tissue. Furthermore, the safety zone between the ligmanetum flavum and the inadvertent dural puncture is smaller in the morbidly obese parturient.[33] Repeated attempts are often associated with increasing risks of regional techniques. Ultrasonography has been suggested to aid the landmark location.[34] Different studies have been published[35–37] to show the usefulness of this technique, especially to identify the midline and the depth of the epidural space.

Post-operatively, obese parturients may require close monitoring. Judicious use of opioids for post-operative pain relief is imperative. A coordinated approach between anaesthetists and obstetricians is vital during the perinatal period to ensure good outcome and patient safety. Early mobilization, thromboprophylaxis, aggressive chest physiotherapy and adequate pain control are cornerstones to successful post-operative obstetric care.[33]

## PATIENTS WITH PREECLAMPSIA

Preeclampsia is a multisystem disorder that is associated with higher maternal morbidity and mortality. For instance, severe preeclampsia could lead to eclamptic seizures and cerebral haemorrhage. The last CEMACH article[6] reports that the majority of parturients who died from preeclampsia died from intracranial haemorrhage. This has led to the recommendation that systolic blood pressure above 160 mmHg requires antihypertensive therapy to avoid cerebral haemorrhage.

Historically, regional anaesthesia was avoided in the severe preeclamptic patient. The main reason for this was because it was postulated that a patient may have profound hypotension following a regional block. Furthermore, fluids administered to treat hypotension would worsen pulmonary oedema.[38] However, it has been found that neuraxial anaesthesia actually provides more stable haemodynamics with fewer swings in blood pressure in the preeclamptic patient.[39] The need for vasopressors during regional anaesthesia in women with severe but haemodynamically stabilized preeclampsia is usually lower compared with healthy parturients.[40] With cautious fluid administration, the risk of pulmonary oedema can be reduced significantly.

Anaesthetists, when performing neuraxial procedures, are often worried about thrombocytopaenia, which may be more common in preeclamptic patients. A lowest platelet count to perform regional anaesthesia has not been identified and is often up to the clinical judgement of each anaesthetist. Most obstetric anaesthetists are comfortable with regional techniques in patients with platelets counts above 70–75,000/mm^3^.[41] Thankfully, spinal epidural haematomas are rare in the obstetric population. A national audit carried out in the United Kingdom of more than 700,000 neuraxial blocks showed only five epidural haematomas. Obstetric patients were included in the audit and none of the haematomas were related to spinal anaesthesia.[42]

General anaesthesia might be the only feasible option when faced with maternal haemorrhage, sustained foetal bradycardia or severe thrombocytopaenia. If general anaesthesia is warranted, the anaesthetist must take precautions to avoid swings in blood pressure, especially during laryngoscopy and airway manipulation, and several intravenous agents have been suggested to attenuate this response, such as labetolol, short-acting opioids, lignocaince, nitroglycerine and continuous infusion of sodium nitroprusside. A difficult airway should be anticipated thus making it vital for the obstetric anaesthetist to be very familiar with failed intubation drills and ensuring that difficult airway equipment is at hand.[41]

## CONCLUSION

There are increasing opportunities in providing anaesthesia for caesarean deliveries. There are new techniques and challenges that the obstetric anaesthetist would need to face. The rate of obesity in developed countries is increasing dramatically, including females in the childbearing age. Obese parturients pose unique challenges as they are associated with preeclampsia, difficulty airway and difficult regional anaesthesia placement. Proseal LMA would be a useful airway device in the management of difficult airway and could see a greater role in an obstetric difficult airway algorithm. Regional anaesthesia may be regarded as superior to general anaesthesia for caesarean section for both the mother and the baby. Regional anaesthesia is performed even for more urgent cases.[43] It would be interesting to see the future development of new medications (suggamadex, levobupivacaine, ropivacaine) and newer techniques (continuous spinal anaesthesia, ultrasound-guided epidural placement). With a greater understanding and further research, obstetric anaesthetists would play an even greater role to optimise the care of the parturient during caesarean delivery.
